# Could Snacks Based on Lupin Be a Nutritious Treat? A Point of View

**DOI:** 10.3390/foods13203227

**Published:** 2024-10-11

**Authors:** Francisco E. Carvajal Larenas, Michael J. Koziol, Mario Caviedes

**Affiliations:** 1 Universidad San Francisco de Quito, Colegio de Ciencias e Ingenierías, Ingeniería en Alimentos, Quito P.O. Box 1712841, Ecuador; 2 Universidad San Francisco de Quito, Colegio de Hospitalidad, Arte Culinario y Turismo, Quito P.O. Box 1712841, Ecuador; mkoziol@usfq.edu.ec; 3 Universidad San Francisco de Quito, Colegio de Ciencias e Ingenierías, Ingeniería en Agronomía, Quito P.O. Box 1712841, Ecuador; mcaviedes@usfq.edu.ec

**Keywords:** nutritious foods, treats, snacks, salty, cereal, lupin

## Abstract

This viewpoint article presents an opinion about snacks made with lupin species. The nutritional quality of cereal-based snacks increased significantly when lupin was added. For instance, the protein and fibre content of lupin snacks could be as high as 55.7% and 8.3% respectively, soluble fibre as high as 61.2% of total fibre, and protein digestibility close to that of casein. As for sensory evaluation, some lupin snacks were ranked even better than controls. Moreover, some lupin snacks had similar or improved rheological behaviour with respect to controls. For instance, expansion indices of 11 versus 3 for controls. In summary, by adjusting formula and processing conditions, it is possible to obtain, at the same time, a healthy and tasty snack with very good machinability and rheological behaviour. This could improve the image and concept of snacks by providing an excellent opportunity for improving the diet quality of habitual consumers of snacks. This point of view also provides suggestions for improving the nutritional, rheological, and sensory evaluation of lupin snacks.

## 1. Introduction

The Global Snack Food Market was expected to reach a USD 638 billon value in 2023 with an annual growth rate of about 5.8%, which is a huge market [1]. Unfortunately, traditional cereal-based snacks [2] are generating a growing worldwide concern because of their high carbohydrate and low protein content, which has been associated with malnutrition, increases in the prevalence of overweight and obesity, and hence the need to consume healthier alternatives [3]. However, in order to be accepted, the new, healthier snacks should be tasty and sustainable [4,5]. In this context, the four major lupin species (*L*. *albus*, *L*. *luteus*, *L*. *angustifolius*, and *L*. *mutabilis*) have an outstanding nutritional composition; i.e., on a 100 g dry basis, they contain 33.9–43.3 g protein; 5.5–18.9 g fat; 8.2–16 g fibre; 3.0–3.9 g ash [6]. As for the fat quality, about 80% of that is unsaturated; i.e., for 100 g fat, 29.1–53.3 g oleic acid, 18.8–49.1 g linoleic acid, and 3.0–9.9 g linolenic acid are contained [6]. Lupins are also eco-friendly; i.e., they produce only 0.3–0.5 t CO_2_ equivalent/ha and reduce the use of fertilizers and pesticides [7]. In addition, lupins are less expensive than meat [8,9,10,11], and some varieties cited present good taste profiles [12], fulfilling the requirements of a healthier and sustainable food [3,12]; for example, Kohajdová et al. [13] mentioned that lupin chips have been proposed as new products in “The Food Ingredients Europe”. Nevertheless, such lupin snacks have barely been investigated. In addition, the global production of lupins, according to the latest data from the FAO [14], is 1.07, 1.42, and 1.64 million tons in the years 2020, 2021, and 2022, respectively, showing an increasing pattern of production. Therefore, this critical point of view is directed at questions concerning the quality of lupin snack products, focusing attention on their chemical composition and nutritional, sensorial, and instrumental evaluation.

## 2. Methods

The information used in this point of view was obtained by three methods. First, by searching the Scopus database and for the text “lupin snacks” and “lupine snacks”, we obtained 28 hits and 12 hits, respectively; in the last dated of search of 13 August 2024, and by searching the Web of Science database with the same keywords and date, we obtained 27 hits in both cases. Then, duplicate information, as well as papers with a different scope than that which is analyzed in this paper, were excluded. Finally, additional information was obtained by checking the bibliographies of the previously selected papers and databases of the authors of this point of view paper. All documents used to support this point of view are part of the scientific databases (peer-reviewed) and most were published within the last 5 years, some within the last 10 years, and a few key papers were older.

## 3. Results and Discussion

### 3.1. Protein

Information about snacks made with lupin species is relatively scarce, although some information can be inferred from that which is available. Thus, the addition of lupins to a cereal base mixture (wheat, maize, or/and rice) increased the protein, fibre, and ash content, while the total carbohydrate contents decrease (Table 1). The fat content shows either increases or decreases. The magnitude of increases in protein, fibre, and ash content depends on the amount of lupin used and if it is used as flour, concentrate, or isolate (Table 1). For example, the use of isolates increases the amounts of protein in mixtures for extrusion by 30, 50, or even 70% [15], which is much higher in comparison to the protein content (17.8%) of extrudates made with mixtures of lupin flour and cereal flours. Nevertheless, the protein content of 17.8% in lupin flour snacks is more than twice the protein content of traditional extrudes made with maize flour (8.1%) or rice flour (7.5%). Moreover, extrudates with 30, 50, or 70% protein content are about 4, 6, and 9 times higher than traditional extruders (control), which are outstanding improvements. Finally, the 50% protein content mixture to be used in extrudates had almost the same protein content as that of a crunchy snack made with 100% *Lupinus mutabilis* (Table 1).

As for the protein quality, extrusion would break cell walls and thus modify protein status [15,16] by destroying covalent and non-covalent bonds [17]. This change would occur in three steps. First, in the cooking zone of the extruder, protein will swell, dissolve, and unfold. Then, new covalent bonds will be formed and finally, in the cooling die, hydrophobic and electrostatic interactions will occur. The net results would be a change in the three-dimensional protein matrix, turning it into a fibrous structure [18] because of the new bond arrangement [17]. This insight is important because it occurs in three of the main species of lupin: namely, *L. luteus*, *L. albus,* and *L. angustifolius*. In addition, extrusion and previous processes (such as soaking and cooking) would also deactivate enzymes [19] and reduce antinutrient contents, i.e., phytic acid, tannins, alfa amylase and trypsin inhibitors [20] that would make the lupin protein more digestible [19], Moreover, extrusion and mixture conditions (temperature, screw speed, and humidity) would also influence the protein structure [18] and product quality [16]. Finally, the combination of lupins with cereals is known to improve the protein quality by complementation [15,21] because pulses like lupin are rich in lysine, but deficient in the amino acids methionine and cysteine (sulphur-containing amino acids) [15]. The final result of all these factors would affect the in vitro digestibility of proteins [19].

**Table 1:** Chemical composition of products made with lupin by-products.

Product	Moisture	Protein	Ash	Fat	Carbohydrates	Fibre	Reference
	%	%	%	%	%	%	
**Extruded**							[15]
30%P (BLPI + WS) ^1^	8.3	30		2.8	67.0 *		
30%P (WLPI + WS) ^1^	7.0	30		2.8	64.1 *	n.a.	
50%P (BLPI + WS) ^1^	7.8	50		3.4	46.7 *	n.a.	
50%P (WLPI + WS) ^1^	6.5	50		3.3	41.8 *	n.a.	
100% MF (Control) ^2^	5.6	8.1	1.6	5.6	81.4	1.6	[1]
90% MF + 10% LAF ^2^	6.9	10.7	1.9	3.2	75.4	1.9	
85% MF + 15% LAF ^2^	7.0	13.7	1.9	4.8	70.2	2.3	
80% MF + 20% LAF ^2^	6.9	17.8	2.1	5.7	64.9	2.6	
Control (100% RF) ^3^	4.6	7.5	0.4	0.2	90.4	1.5	[21]
90% RF + 10% SPF ^3^	4.3	7.8	0.5	0.3	89.4	2.1	
87.5% RF + 10% SPF + 2.5% LAF ^3^	4.3	8.6	0.6	0.4	88.3	2.2	
85.0% RF + 10% SPF + 5.0% LAF ^3^	4.3	9.4	0.7	0.4	87.2	2.2	
82.5% RF + 10% SPF + 7.5% LAF ^3^	4.2	10.1	0.8	0.5	86.2	2.3	
80.0% RF + 10% SPF + 10.0% LAF ^3^	4.2	11.1	0.9	0.6	85.0	2.4	
100% LMF ^4^	n.a.	55.7	1.7	24.8	17.8	n.a.	[19]
**Dried and fried snacks**							
100% LM ^5^	6.0	51.7	2.4	21.6	15.9	8.3	[22]

^1^ **Extruded** 30 or 50% protein content with blue lupin (*Lupinus angustifolius* cv. Boregine) protein isolate (**BLPI**, 89.8% protein, 2.7% lipids), wheat starch (**WS**, 67.9% starch, 15.5% protein, 4.9% lipids), 2% oil and 0.5% salt. **Extruded** 30 or 50% protein content with white lupin (*Lupinus albus* cv. Butan) protein isolate (**WLPI**, 89.7% protein, 2.3% lipids), wheat starch (**WS**), 2% oil and 0.5% salt. * As starch. ^2^ Maize flour (**MF,** 70.8% carbohydrates, 10.5% moisture, 10.2% protein, 4.3% fat, 2.2% ash, 2% fibre, carbohydrates by difference), *Lupinus albus* flour (**LAF**, 39.9% protein, 32.5% carbohydrates, 14.5% fat, 7.8% moisture, 2.6% ash, 2.6% fibre), ^3^ rice flour (**RF**, 91.36% carbohydrates, 9.42% moisture, 7.71% protein, 0.22% fat, 0.25% fibre, 0.68% ash), sweet potato flour (**SPF**, 91.41% carbohydrates, 6.25% moisture, 3.97% protein, 4.4% fibre, 2.13% ash, 0.74% fat), *Lupinus albus* flour (**LAF** 51.43% carbohydrates, 40.37% protein, 8.40% moisture, 3.99% fibre, 3.18% fat, 1.25% ash). **Micronutrients (mg/100 g), control**: Mg, 0.0; Ca, 20.14; P, 0.0; Fe, 1.67; Zn, 0.52. **90% RF + 10% SPF**: Mg, 0.0; Ca, 29.5; P, 0.0; Fe, 1.88; Zn, 1.35. **80% RF + 10% SPF + 10% LAF**: Mg, 12.26; Ca, 29.78; P, 0.05; Fe, 2.17; Zn, 1.66. ^4^ **LMF** *Lupinus mutabilis* flour. ^5^ Fried snack 100% **LM** (*Lupinus mutabilis* Sweet, 51.7% protein, 21.6% fat, 8.3% fibre, 2.4% ash, 15.9% carbohydrates, 6.0% moisture). **Micronutrients (mg/100):** Na, 40; K, 20; Mg, 160; Ca, 500; P, 430; Fe, 9.9; Zn, 5.4.; Mn, 2.4; Cu, 0.9.

Thus, the protein digestibility of *Lupinus angustifolius* increased from 78.2% in the raw material mixture (lupin isolate: concentrated 50:50) to 80.94–85.94% in the extruded mixture (extrusion conditions: water feed 55–68%, temp 180–155 °C, 1200 rpm) [18]. Similarly, in *L*. *mutabilis*, digestibility changed from 63.7% (debittered flour) to 68.1% (extruder flour, protein content 55.7 g/100 g) (extrusion conditions: 35% humidity, 140 °C) [19]. The product also showed minimal heat damage (extruded = 8.7 mg furosine/100 g of protein vs. debittered flour = 10.52) [19]. The difference in protein digestibility is similar to that reported by other authors for other lupin species and other grains. Thus, Palanisamy et al. [18] found that in vitro digestibility of lupin extrudates was 80.9–85.9%, compared with 78.2% in raw materials. Similarly, Abd El-Hady et al. [20] reported in vitro protein digestibility for raw vs. extruded kidney bean changed from 70.6 to 79.3%, from 74.0 to 81.1% in chickpeas, and from 74.5 to 78.0% in peas. Extrusion seems to improve protein digestibility [23]. However, these improved values are still lower than those of casein protein isolate, 87.1%, although a digestibility of 85.9% [18] for extruded lupin closely approximates this value.

In addition, even though most of authors agreed with the idea than extrusion improves the protein digestibility of extrudes, this improvement would depend on extrusion conditions. For example, Palanisamy et al. [18], found that addition of water between 40 and 55% showed no significant differences in protein digestibility, whereas an addition between 60 and 68% improved digestibility significantly. The apparent reason is that reduced protein aggregation would enhance digestibility. In fact, either too little water (40%) or too much (68%) would not favour the formation of a fibrous network structure, because high moisture would reduce denaturation and protein interactions, and as a result lower cross-linking. On the other hand, low moisture content results in low cross-linking because the protein is not completely hydrates. Thus, the water feed during extrusion is critical [18].

Apparently, increasing the fibrous network structure has a cost in terms of protein digestibility. Temperature also affects the denaturalization of lupin proteins. For example, Martin et al. [15] reported endothermic peaks (associated with protein denaturalization) at 86.31 °C for white lupin and 96.15 °C for blue lupin. Increasing barrel temperatures to 155–180 °C would diminish protein digestibility due to increases in non-enzymatic browning [18].

As for the effect of screw speed (rpm) and particle size, increasing the speed would produce more mechanical energy, which in turn would cause an increment in temperature and corresponding protein denaturation dependent upon the temperature reached [18]. Particle size is also important, as smaller particles will present a greater surface area available for link creation [15,18,24]. For example, the use of a mesh with a particle size of 2 mm improved in vitro digestibility [19].

### 3.2. Fibre

Regarding fibre content, the addition of lupin would both increase the fibre and change the fibre properties of extrudates [19]. However, only snacks based on 100% lupin had a fibre content of 8.3%, which is more than three times the fibre content of other snacks containing lupin (Table 1). Moreover, the extrusion process seems to convert part of the insoluble fibre into soluble fibre [24,25]. For example, the study by Naumann et al. [26] on the *L. angustifolius* cv Boregine kernel (extrusion conditions: 150 °C, moisture content 20%, speed 200 rpm, particle size < 500 um, energy used: 2.47 kw-h/kg) showed that with respect to total dietary fibre, soluble fibre was increased from 2.3% in the raw material to 61.2% in the extruded material. This value is much higher than the 9.82% reported by Zhong et al. [24] (extrusion conditions: 140 °C, 35% moisture content, 400 rpm, <500 um particle size, 0.22 kw-h/kg) or the 11.07% reported by Zhong et al. [25] (extrusion conditions: 135 °C, 26.6% moisture, speed 350 rpm, <500 um particle size, 0.34 kw-h/kg). This important difference in the proportion of soluble fibre perhaps could be explained by Zhong et al. [25], who established a quadratic model which showed that the main aspect effecting an increase in the content of soluble dietary fibre is a reduction in moisture (*p* < 0.0001), followed by high temperatures (*p* = 0.0053), with no effect from the barrel speed alone. A higher amount of soluble fibre seems to be achieved with a moisture content of 20% and a temperature of 150 °C, which are the conditions used by Naumann et al. [26], but not by Zhong et al. [25] and Zhong et al. [24]. On the other hand, working with low moisture contents (20%) and high temperatures (150 °C) would have a high price in terms of energy (about 7–11 times that needed when working at moistures contents of 26.6–35% and temperatures of 135 or 140 °C). This is an important finding resulting from the fortuitous use of *L. angustifolius* in all three studies. As for the functionality of the fibre, its consumption is associated with reduction in colorectal cancer [16,23]. In addition, samples of extruded material digested in vitro showed increases in viscosity, apparently due to solubilization of pectin-like polymers, which in turn generated a reduction in the diffusion of bile acids, associate with lowering cholesterol in blood [26]. Moreover, Zhong et al. [25], found that extrusion cooking of the seed coat of Australian sweet lupin (*Lupinus angustifolius*) (141.1 °C, 30% moisture content, and 400 rpm) had either little or no effect on the bio-accessibility of individual polyphenols. In fact, the 3D surface optimization model proposed by these authors would suggest that higher concentrations of total individual phenolic content in the free phenolic extract (TFIPC) and apinegin-7-O-B-apiofuranosyl-6,8-di-C-B-glucopyranoside (Api-Api*f*-di-Gle*p*) were obtained under higher screw speed, high temperature, and low moisture. This means that the conditions found by Naumann et al. [26] would led not only to the transformation of higher amounts of insoluble fibre to soluble, with all the accruing benefits of soluble fibre, but also that conditions would retain higher antioxidants values. Extrudes obtained by Ramos Diaz et al. [27] in similar conditions also retained total phenolic compounds and folate. This would suggest that under the conditions mentioned, extruded lupin seed coats might have functional properties physiologically.

### 3.3. Fat

Fat content in lupin snacks was found both in low (0.2–5.7%) and high amounts (21.6–24.82%), Table 1. Such variation in fat content could attributed to two factors: the fat content of the ingredients in the mixture, and the processes used to prepare the snacks. Lower fat contents were found in lupin snacks in which the majority of ingredients also had low fat content, i.e., wheat starch (4.9%), maize flour (4.3%), *L. angustifolius* (2.7%), *L. albus* (2.13–2.6%), sweet potato flour (0.74%), or rice flour (0.22%) [1,15,21], whereas higher fat content was found when the product was made with 100% *L. mutabilis* (fat content 21.6–24.8%), the lupin species with the highest content of fat [6]. The processes used to prepare the lupin snacks also influence final fat content. Most extruded lupin snacks have low fat content because, in order to obtain desirable rheological behaviour during extrusion (high expansion index), the starch content must be high [21], thereby reducing the fat content. Other lupin snacks (chips) can be obtained from mixtures that are kneaded and then baked or deep fried [28]. The absorption of oil during frying will result in higher fat content. For example, Özcan et al. [29] prepared snacks with *Lupinus albus* using 22 mixtures of 25–60% lupin flour made from whole or hulled seeds, 30–60% whole-wheat flour, 0–15% corn flour, and 5–10% guar gum. The mixtures were kneaded, moulded, and deep-fried in sunflower oil (180 °C, 1 min) or baked at 180 °C, 15 min). The amount of oil absorbed varied between 14.03 and 22.45% for fried snacks compared with fat contents of 2.60 to 10.03% in the baked snacks.

The absorption of oil in these products is higher than that of a snack made with 100% *Lupinus mutabilis* which was dried prior to deep frying. In this case, the final fat content of the product (21.6%) [22] depends on the fat content of the lupin species (16.6%) [30] and the amount of fat absorbed during deep frying. The amount of oil absorbed for all these lupin snacks is lower than that found in other snacks such as the following: wheat chips (21.5–34.9%), corn chips (22.51–28.28%), commercial potato chips (31%), and commercial baked potato chips (15.9%) [29]. The amount of oil absorbed is directly proportional to the size and distribution of pores in the matrix structure of the snacks. Techniques such as pre-drying and using hydrocolloids are reported to reduce oil absorption [29]. The incorporation of guar gum in the *Lupinus albus* snacks and the pre-drying of the *Lupinus mutabilis* snack prior to deep frying could partially explain the decreased oil absorption.

The fatty acid profiles of the 22 *Lupinus albus* snacks which were deep-fried were [29]: oleic acid (30.19–52.44%), linoleic acid (24.04–56.44%), linolenic acid (0.05–0.86), palmitic acid (6.73–12.44%), and stearic acid (4.26–7.25%). The baked snacks had the following: oleic acid (30.43–52.84%), linoleic acid (19.77–54.12%), linolenic acid (0.05–0.68%), palmitic acid (7.01–13.75%), and stearic acid (4.74–8.53%).

The fatty acid profile of unsaturated fatty acids in *Lupinus albus* is as follows: 53.6% oleic acid, 18.8% linoleic acid, and 8.9% linolenic acid [30] (I17). The snacks presented lower concentrations of oleic and linolenic acids but higher concentrations of linoleic acids. This is probably a reflection of the effects of other ingredients used in the formulation of the snacks.

Finally, lupin snacks can be engineered to present high, medium, or low fat contents according to what the market dictates. Extruded snacks with low fat content and good expansion indexes can be further modified to present higher fat contents (when needed) by frying or using a drenching system. Similarly, snack formulas and processing conditions can be manipulated to regulate the fat content of kneaded products. For example, baked low-fat mixtures can have their fat contents increased by frying or drenching.

### 3.4. Micronutrients

As for the micronutrient content, the addition of *L. albus* flour (2.5–10%) to extrudates increased the ash content by 0.57–0.90% when compared with control (90% rice + 10% sweet potato; ash content 0.47%) [21]. The addition of whole *L. albus* flour would be better than the addition of de-hulled *L. albus* flour. Baking would be more advantageous than deep frying [28]. For example, in chips made with 40–45% whole-lupin flour, baked chips showed the highest content of calcium and iron (4066 and 166.50 mg/kg chip, respectively) when compared with fried chips (2423 and 100.10 mg/kg chip, respectively). Similarly, in chips made with 40–45% de-hulled lupin flour, baked chips also showed higher calcium and iron contents (3143 and 134.30 mg/kg chip, respectively) compared with fried chips (2408 and 66.80 mg/kg chip, respectively) [28]. This implies that the lupin hull would have a higher ash content compared with the lupin endosperm. Thus, Zhong et al. [25] reported that 67.5% of the calcium was located in the seed hull of *L. angustifolius*. In addition, the deep frying process could cause a leaching of minerals and/or the absorption of fat, thus causing a dilution of the ash content. As for the impact of the ash increment in daily intake, it depends on the formula and process used. For instance, one portion of 100 g of extruded snack (80% rice flour + 10% potato flour + 10% *L. albus* flour) contained 29.78 mg Ca, 2.17 mg Fe, and 1.66 mg Zn, which represents 2.27%, 27.13%, and 20.75%, respectively, of the recommended daily requirements of calcium (1300 mg), iron (8 mg), and zinc (8 mg) for children (male or female) 9–12 years old [21]. When comparing this formula with control (extruding 90% rice, 10% sweet potato) the mineral content is almost the same: Ca 29.48 mg; Fe 1.88 mg; and Zn 1.35 mg) [21]. However, in baked snacks (40–45% whole lupin flour + 38–45% whole-wheat flour + 5–14% corn flour+ 5–8% guar gum) the calcium, iron, and zinc content were 406.6, 13.4, and 2.3 mg/100 g respectively (representing 31.2, 167.5%, and 28.8% of the daily requirement for children (male and female) 9–12 years old, an important improvement especially regarding calcium and iron [28]. Special attention should be paid to check if the increase in iron content is not an artefact of contamination during extrusion cooking [25]. As for the minerals’ bio-accessibility (% of material released) and bioavailability (% of material absorbed), Zhong et al. [25] found that extrusion (120–160 °C, moisture content 26–40%, 300–400 rpm) showed no significant effect on either parameters for iron, copper, zinc, or magnesium even though the total individual polyphenol content, and hence their ability to ligate minerals, decreased between 14 and 27% after extrusion (compared with raw material). In addition, it is expected that the amount of minerals in extruded products will relay also on the lupin variety and on the technology used to debitter lupin and prepare flour, concentrated or isolated [6,31,32]. The addition or supplementation of new materials can also be considered. For example, the addition of ferritin and leghaemoglobin has been reported to increase the iron content [33].

### 3.5. Functional Properties

Some pharmaceutical activity [16] has been suggested for pulses like lupin seeds, such as hypocholesteraemic activity or a reduction in postprandial glucose levels [22,32,34], reduction in systolic or diastolic blood pressure [22], and risk reduction for developing colorectal cancer [23].

Moreover, a reduction in metabolic syndrome factors in overweight and obese adults is reported to be associated with regular consumption of pulses for eight weeks [35]. Although the evidence on these subjects is very important and encouraging, more confirming research is needed.

### 3.6. Instrumental Evaluation

Regarding instrumental evaluation of lupin snacks, studies show that formula, pre-treatment, and processing conditions have a direct impact on bite quality [5,36]. For example, the expansion index depends on the content and quality of carbohydrates (starch and fibre), protein, moisture, and salts present in the mixture to be extruded, as well as the processing conditions (Table 1 and Table 2) [21,24,26,37]. Thus, Muhammed et al. [1] studied mixes of maize flour (80–100%), lupin flour (0–20%), barrel temperatures (120–150 °C), and moisture content (14–18%), and found the expansion index to be between 0.7 and 1.2 with a direct relationship with temperature and an inverse relationship with the amount of lupin (protein content) and moisture content. The most influential parameter was the lupin content. Increasing the amount of lupin flour (protein), resulted in a reduction in maize flour (starch) and in the expansion index, as higher expansion indexes are associated with higher amounts of starch [21]. On the other hand, another study by Martin et al. [15] also reported effects of formula (up to 50% protein content) and processing conditions (pre-treatments and additives) on expansion indices, but this time with a very different outcome (expansion index between 4 and 12; all mixtures were adjusted to 33% moisture content and extruded at 160 °C). A third study by Algarni et al. [21] showed expansion values between those of the studies of Muhammed et al. [1] and Martin et al. [15], reporting expansion values which varied between 2.4 and 3.2. The composition of all mixtures studied are presented in Table 2. Several factors might explain differences in the outcomes. First, it is possible to obtain extrudates with both high protein content (30–50%) and a satisfactory expansion index (4–12). However, in order to obtain this result, it is necessary to use protein isolates instead of lupin flour because the addition of isolates significantly increases the amount of protein in the mixture with a minimal impact on the starch content and therefore on the expansion index. Second, the inclusion of a pre-treatment (30 min at 80 °C plus 10 min at 120 °C) and additives (2.0% oil and 0.5% NaCl) to the mixtures before extrusion would increase the expansion index, perhaps by denaturalization of proteins (pre-treatment), and perhaps by an effect of lubrication (oil) and variation on ionic strength (NaCl). However, these effects would only be effective at protein contents up to 30% in the mixtures (expansion index 10–12). In the case of mixtures with protein content of 50% (with or without pre-treatment and with or without additives) expansion index of (4–4.5) are reported. For this amount of protein (50%) the negative effect of reduction in the starch content would be higher than the positive effect of the pre-treatments and/or additives. In fact, for mixtures with 30% protein content, neither pre-treatment nor the inclusion of additives resulted in an expansion index (6–7) higher than mixtures 50% protein with pre-treatment and with additives. Third, the type of lupin protein could also affect the expansion index. Mixtures, with 30% protein content, of blue and white lupin showed expansion indexes of about 6 and 7 respectively, and these values increased to 10 for white and to 12 for blue lupin when pre-treatment and additives were included [15]. Moreover, these authors also studied mixtures (30% and 50% protein content) of other pulses (lentil and faba bean) simultaneously with blue and white lupin (changing nothing more than the pulse). In all cases the expansion indices of lentils and faba bean were lower than those of lupins, emphasizing the need to study not only the protein content but also its composition. Fourth, the type of starch might also affect the expansion index [38,39]. Thus, Muhammed et al. [1] used maize flour (starch), Algarni et al. [21] used rice flour, and Martin et al. [15] used wheat starch. Ratios of amylose to amylopectin and their composition could be important. Fifth, authors Muhammed et al., Martin et al. [1,15] also agreed that the expansion index would be likewise affected by moisture content. Thus, Muhammed et al. [1], working with moisture content of 14–18%, found an inverse relationship. On the contrary, Palanisamy at al. [18] found that denser structures (lower expansion index) are associated with reduced moisture content (direct relationship), which would agree with the results of Martin et al. [15] working with 33% moisture content and obtaining the highest expansion index. Therefore, it seems that mixtures with high moisture content have more free water that can be evaporated, thus facilitating a higher expansion index. In addition, a difference in moisture content between 14 and 18% as used by Muhammed et al. [1] is perhaps too small to see the real effect of this variable.

Summarizing, the expansion index of the extrudate would be influenced not only by the protein, starch, and moisture content, and inclusion of additives in the matrix, but also by the quality (composition and structural functionality) of all components of the matrix, as well as the processing conditions [27] and pre-treatments used. For example, the use of methanol for fat extraction of concentrated lupin flour would exclude apolar lipids (triglycerides), while the use of *n*-hexane would exclude polar lipids (fatty acids and phospholipids), having an opposite effect on the functionality of those lupin by-products [13]. Moreover, considering that protein isolates and concentrates are obtained by different methods such as aqueous (alkaline) extraction combined with isoelectric precipitation or ultrasound, by ultrafiltration, by assisted protein extraction, or by dry fractioning [15,16], the effect of these treatments on the functional behaviour of protein concentrates or isolates within the extrusion matrix for snack products is unknown. Finally, the type and amount of hydrocolloid used might affect the texture of snacks [28] since they have different behaviours [40,41].

The expansion process within the extruder needs energy, and this is reported to be as low as 0.13 kw-h/kg [24] or as high as 2.81 kw-h/kg [26]. Lower energy consumption in the extruder [24] has been correlated with high moisture content (*p* < 0.001, r = 0.77). This tendency can also be observed in data of Martin et al., Zhong et al., and Naumann et al. [15,25,26]. High moisture content would need less torque and, therefore, less energy to move the mixture through screws. Another factor that influences energy consumption is screw speed, but this time, in a direct relationship [25,26]. Interestingly, increasing the barrel temperature seems to slightly reduce the energy used in extrusion. This outcome might be because the energy invested to increase the temperature and thus facilitate the movement through the extruder by reduction in viscosity will be lower than the energy saved by torque reduction. Data from Naumann et al. [26] and Zhong et al. [25] seem to support this affirmation, Table 2. Finally, the energy used in extrusion is, perhaps, also related to extruder design, formula, and particle size, variables which merit further study.

**Table 2:** Sensorial and/or instrumental evaluation of snacks made with lupin by-products.

Used in	Formula and Conditions	Instrumental Evaluation	Sensory Evaluation	Reference
Ext ^1^	30%P (BLPI + WS)	SEI ≈ 11; D ≈ 0.45; SH ≈ 0.25	S = highest preference	[15]
Ext ^1^	30%P (WLPI + WS)	SEI ≈ 9.5; D ≈ 0.55 SH ≈ 0.25	S = Good evaluation	
Ext ^1^	50%P (BLPI + WS)	SEI ≈ 5; D ≈ 0.6 SH ≈ 0.7	S = highest preference	
Ext ^1^	50%P (WLPI + WS)	SEI ≈ 5; D ≈ 0.6; SH ≈ 0.5	S = Good evaluation	
Ext ^2^	100% MF.	RER = 0.92; BD = 0.58; WAI = 5.8; WSI = 10.2	C = 4.9; Ta = 5.6; F = 5.8; Te = 5.7; OA = 5.5	[1]
Ext ^2^	90% MF + 10% LAF; T = 135 C; M = 18%	RER = 0.89; BD = 0.65; WAI = 6.3; WSI = 11.5	C = 6.2; Ta = 6.2; F = 6.3; Te = 6.0; OA = 6.2	
Ext ^2^	85% MF + 15% LAF; T = 120 C, M = 18%	RER = 0.95; BD = 0.53; WAI = 5.6; WSI = 12.3	C = 7.2; Ta = 6.2; F = 6.5; Te = 6.5; OA = 6.6	
Ext ^2^	85% MF + 15% LAF; T = 150 C, M = 14%	RER = 1.2; BD = 0.33; WAI = 5.7; WSI = 15.3	C = 6.7; Ta = 6.2; F = 6.7; Te = 6.4; OA = 6.7	
Ext ^2^	80% MF + 20% LAF; T = 120 C, M = 16%	RER = 0.71; BD = 0.8; WAI = 4.9; WSI = 13.8	C = 7.3; Ta = 6.2; F = 6.2; Te = 6.1; OA = 6.8	
Ext ^3^	100% RF	ER = 3.21; BD = 0.77 **; WAI = 9.7; WSI = 23.7	C = 8.7; Ta = 9.6; O = 9.6; Cr = 9.3; AT = 5.6; OA = 8.5	[21]
Ext ^3^	90% RF, 10% SP	ER = 3.19, BD = 0.78 **; WAI = 9.6; WSI = 23.5	C = 9.7; Ta = 9.6; O = 9.7; Cr = 9.6; AT = 5.6; OA = 8.8	
Ext ^3^	87.5% RF, 10% SPF, 2.5% LAF	ER = 3.11; BD = 0.80 **; WAI = 8.5; WSI = 22.3	C = 9.6; Ta = 9.6; O = 9.7; Cr = 9.6; AT = 5.6; OA = 8.9	
Ext ^3^	85.0% RF, 10% SPF, 5% LAF	ER = 3.0; BD = 0.84 **; WAI = 8.1; WSI = 20.8	C = 9.6; Ta = 9.6; O = 9.7; Cr = 9.7; AT = 5.7; OA = 8.8	
Ext ^3^	82.5% RF, 10% SPF, 7.5% LAF	ER = 2.95; BD = 0.87 **; WAI = 7.8; WSI = 18.8	C = 9.5; Ta = 9.7; O = 9.8; Cr = 9.8; AT = 6.2; OA = 8.9	
Ext ^3^	80.0% RF, 10% SPF, 10% LAF	ER = 2.42; BD = 0.90 **; WAI = 7.2; WSI = 16.6	C = 9.5; Ta = 9.4; O = 9.5; Cr = 9.4; AT = 6.32; OA = 8.7	
(D + F) ^4^	100% LM		S = 100% judges accepted, 83% perfect adherence	[22]

Ext ^1^ = Extruded. 30 or 50% **P** (protein content) with **BLPI** = blue lupin (*Lupinus angustifolius* cv. Boregine) protein isolates + **WS** = wheat starch. (**WLPI** = white lupin (*Lupinus albus* cv. Butan) protein isolates. Extrusion (constant) conditions: 2% oil and 0.5% salt. Mixtures preheated for 30 min at 80 °C and 10 min at 120 °C. Extrusion at 160 °C = 160 °C, Moisture 33%, 300 rpm. Results: **SEI** = specific expansion index (times die diameter); **D** = density (g/cm^3^); **SH** = specific hardness (N/mm^2^). Sensory evaluation (**S**) by 10–15 trained judges (24–54 years); aroma profile analysis by ISO 13299:2016 [42]. Ext ^2^ **MF** = maize flour (cv. BH-540, 0.5 mm mesh), **LAF** = *Lupinus albus* flour (0.5 mesh), Feeding parameters (60 g/min and 200 rpm); **T** = barrel temperature at zone 3; **M** = moisture. **RER** = radial expansion ratio (ratio extrudate/ratio die, equivalent to SEI). **BD** = bulk density (BD g/cc). **WAI** = water abortion index (g/g); **WSI** = water solubility index (%); 50 semi trained judges, 9-points scale: **C** = colour; **Ta** = taste; **F** = flavour; **Te** = texture; **OA** = overall acceptability. Ext ^3^ **RF** = rice flour (400–600 microns). **SPF** = sweet potato flour < 315 microns); **LAF** = *Lupinus albus* flour (<60 mesh). Extruded constant conditions: T = 200 °C, 249 rpm. Results: **ER** = expand ratio (extrudate diameter/die diameter, equivalent to SEI or RER); ** = transformed from the original values to compare. Sensory evaluation according to Ibrahim et al. [43]; **O** = odour; **Cr** = crispness (≈texture); **AT** = aftertaste. **(D + F)** ^4^ (Dried and fried snack); **S**, 64 untrained judges. A total of 10 g consumed daily for 14 weeks.

As for the water absorption index, increasing the protein, fat, and fibre contents, and at the same time reducing the starch content, shows a tendency to diminish the water absorption index (Table 1 and Table 2). The magnitude of that relationship would depend upon the amount of those materials in the product jointly with the impact of isolation methods [16], thermal treatment, pH, ionic strength, amino acid profile, hydrophobicity, and spatial conformation of the matrix [6,30]. As for the water solubility index, increasing the protein content in the formula resulted in two different outcomes in the extruded product, i.e., increases [1] and reductions [21]. This difference in the outcome might be influenced by (a) the type of starch used [44], Table 1 and Table 2, and (b) balance of hydrophilic–hydrophobic amino acids on the surface [16] and the conditions of the mixture (temperature, pH, and ionic strength) [6].

Since both products were extruded at a high temperature [1,21], denaturation of proteins is expected in both cases. However, as pH and ionic strength of extrudates remain unknown it is impossible to elucidate the effect of these variables on protein solubility. Moreover, particle size could also affect the properties of materials, such as its water and oil binding capacity, because smaller particles will present a greater surface area available for creating links [15,18,24]. Density and hardness of extrudes show an inverse relationship with the expansion index (Table 2).

### 3.7. Sensory Evaluation

The use of high cooking temperatures (120–160 °C) reduces the beany flavour of lupin extrudates, which enhanced the sensorial scores [1]. Thus, mixtures of legumes (blue or white lupin, faba bean, or lentils) and wheat starch with 30–50% protein content were extruded at cooking temperatures of 140–160 °C and analyzed by 10 or 15 trained judges. They concluded that blue or white lupin extrudates were preferred over the extrudates of faba bean or lentil, giving the impression that the lupin snacks would be saltier and sweeter [15], and some lupin extrudates were rated even higher than control (without lupin). For example, Algarni et al. [21] evaluated color, taste, odour, crispness, aftertaste, and overall acceptability of four extrudates containing 2.5, 5.0, 7.5, or 10% *Lupinus albus* flour and 10% sweet potato flour, with broken rice flour added to achieve 100%. These were compared with two control extrudates (100% broken rice flour or 90% broken rice flour with 10% sweet potato flour). Results showed that, in general, lupin extrudates rated better than controls, with the sample prepared with 7.50% lupin flour being preferred in taste, odour, crispness, and overall acceptability. Colour was rated higher in extrudates containing 2.5% lupin flour and aftertaste was rated higher in extrudates containing 10% lupin flour (Table 2). Similarly, Muhammed et al. [1] evaluated colour, flavour, texture, taste, and overall acceptability of three extruded mixtures containing 10, 15, or 20% *Lupinus albus* flour and 90, 85, or 80% maize flour (BH-540 variety) and compared them with an extruded control (100% maize flour). The results obtained from 50 semi-trained judges (19–50 years old, male and female) showed that all lupin extrudates rated much better than the control. It is valid to note that the mixtures with 15% *Lupinus albus* extruded at 150 °C were rated slightly better than other mixtures (Table 2). In addition, fried lupin alone (*L. mutabilis*) or combined (*L. albus*) with cereals is reported to have had good sensorial acceptance [22,29,45]. Özcan et al. [29] evaluated taste, crispness, colour, odour, oiliness, and overall acceptability of mixtures containing whole-lupin flour (30–60%) or hulled lupin flour (30–60%), whole-wheat flour (35–55%), corn flour (0–15%) and guar gum (5–10%), and compared them with controls (60–70% corn flour and 30–40% whole-wheat flour). Kneaded mixtures were baked at 180 °C for 15 min or deep fried at 180 °C for 1 min using sunflower oil. Results of the evaluation of ten well-trained judges using a 5-point hedonic scale showed that deep frying rated better than baking for all mixtures. One of the better mixtures was that which was deep fried and contained 30–35% whole lupin flour, 50–55% whole-wheat flour, 5–10% corn flour and 5–10% guar gum, which was rated higher in taste (3.4), crispiness (3.9), colour (3.8), and overall acceptability (3.5) when compared with the deep-fried control (3.2, 3.5, 3.2, and 3.4, respectively). Baked lupin mixtures were rated better than the control; however, almost none of the samples attained a score of 3, emphasizing not only the importance of the formula but also of the processes used.

The influences of lupin processing, such as debittering, could also influence the acceptance of the final product. Seeds of *Lupinus albus* [46] were debittered by boiling for 75 min followed by washing for 144 h using three different methods, namely with water at about 25 °C, with 0.5% sodium bicarbonate at 25 °C, and with hot water at 65 °C. Seeds were immersed continuously in the debittering solvents, which were changed every 12 h. Finally, the debittered seeds were keep in a brine of 6% NaCl for 12 h. The sensory evaluation (appearance, colour, odour, texture, taste, bitterness, and overall acceptance) was performed by six trained panellists using a 5-point hedonic scale. For the water process, all characteristics ranked between 4.2 and 4.6; for the 0.5% sodium bicarbonate process all characteristics ranked between 3.9 and 4.1, and for the hot water process (65 °C) all characteristics ranked between 1.4 and 2.7. The overall scores were 4.6, 3.9, and 2.1, respectively. These results illustrate the relationship between debittering processes and sensory evaluation. This is in agreement with other studies that show that processes such as blending [13], roasting [6] or 3D printing [47] can affect the sensory or rheological evaluation.

Lupin added to snack mixtures could improve the micro-distribution of water, and reduce the mass shrinkage, while at the same time improving crispness. Roasting will develop a nutty flavour, which could enhance acceptability [6]. Cold 3D extrusion printing of lupin mixtures followed by baking at 180 °C for 15–30 min can produce snacks with a harder and more crumbly texture compared with the same mixtures conventionally processed (put in square moulds and then baked under the same conditions). Apparently, the 3D printing process produces larger air gaps within the microstructures that lead to higher moisture loss, thus resulting in a denser structure [47].

## 4. Feasibility of Lupin Snacks from a Global Perspective

The addition of lupin (flour, concentrate, or protein isolate) to cereal-based snacks improves their nutritional quality, partially replacing the cereals normally used, mainly maize (corn), wheat, and rice. The latest data available from the FAO [14] show that the world’s production of these cereals in millions of tons in 2022 was 1163.5 for corn, 808.4 for wheat, and 776.5 for rice, while the production of lupin in the same year was 1.6 million tons. The current production of lupin is still marginal in comparison with that of the main cereal crops, and it would therefore seem that its incorporation into all products would be impossible, but it could be used for partial substitution in snacks. Data from the FAO [48] concerning the apparent consumption of savoury snacks worldwide varies from 0 to 3.5 g/capita/day. Considering a global population of eight billion consumers of snacks and four different consumption levels of snacks, namely 0.5, 1.5, 2.5 and 3.5 g/capita/day, the respective total amounts of savoury snacks consumed worldwide per year would be 1.46, 4.38, 7.30, and 7.30 million tons. Thus, the production of raw lupin in 2022 would represent 112.6, 37.6, 22.53, or 16% of the total production of savoury snacks. Taking into consideration that lentils, beans, faba beans, and even soya beans might be used in the elaboration of savoury snacks, the yearly production of lupin need not be considered as limiting in regard to its use. Obviously, this does not preclude the desirability of increasing the production of lupin.

The production of lupin generates the lowest environmental impact in terms of global warming, i.e., 0.57 kg CO_2_ equivalent/kg raw material [49], compared with maize, wheat, rice, or beef with 1.0, 1.4, 4.0, or 60 kg CO_2_ equivalent/kg [50]. In addition, the incorporation of legumes in crop rotation prevents pest infestations, thus reducing the use of pesticides, and fixes atmospheric nitrogen problems, thus reducing the use of fertilizer. Such systems of crop rotation can improve yield, protein content, and grain quality in successive cereal crops. Rabolledo-Leiva et al. [7] incorporated lupin into an organic crop rotation system. The study lasted six years and found that rotating lupin with wheat, potato, oilseed rape, or maize produced emissions of about 0.3–0.5 for lupins, 0.5–0.6 for wheat, 1.2 for potato, 0.2–1.3 for oilseed rape, and 2.1 for maize in terms of tons of CO_2_ equivalent/ha. The introduction of lupin as a rotational crop represented a favourable environmental and economic strategy, increasing yields of the subsequent crop and reducing the need for nitrogen fertilizers due to the dependence upon diesel in farm machinery related to the application of fertilizers and pesticides. Therefore, the production of lupin snacks seems to be a feasible economic option which is also ecologically sustainable.

## 5. Conclusions

The addition of lupin species to cereal snacks is highly recommended, because such addition substantially improves the chemical composition and nutritional quality of snacks as regards protein, fibre, and ash content, and when desired, lupins can also improve fat content and quality. The degree of the improvements in the chemical composition of snacks will be dependent on the chemical composition of the lupin species used, as well as the form and quantity in which it is used, i.e., flour or protein concentrate or isolate (Table 1).

It seems possible to develop treats that simultaneously improve the nutritional quality and show good sensorial acceptance.

The use of lupins can contribute positively to the ecosystem.

This could improve the concept and image of snacks and provide an impetus for the further research and development of such improved products.

## 6. Recommendations for New Studies on Salty Lupin Snacks

Increasing the lupin content in cereal-based snacks will require the balancing of various factors: 1. The macronutrient formula (protein, starch, fibre, fat, minerals, and humidity). 2. The quality of those macronutrients (for example, the types of proteins and types of starches used). 3. The chemical conditions of the formula (pH, ionic strength). 4. The processing conditions (temperature, humidity, velocity (rpm)). 5. The hydrocolloid and amount used. 6. The pre-treatments applied to the mixture, such as the germination [51], pre-heating, lubrication, acylation, and/or succinylation processes. 7. The processes used to obtain the lupin by-products (flour, concentrate, isolate) such as soaking, drying, defatting, or using solvents, ultrasound, ultrafiltration, dry fractioning, assisted protein extraction, or milling (particle size), ionic strength and pH [6,16]. Regarding the rheological and textural qualities of salty bites, a twin-screw extruder has commonly been used, although other technologies such as high-pressure or extensional flow could also modify the textures of the final products [11,52]. The use of analysis methods such as Fourier-Transform Infrared (FTIR) Spectra, Confocal Laser Scanning Microscopy (CLSM), X-ray Diffraction (XRD), and Scanning Electron Microscopy (SEM) can helpful to visualize, understand, and improve the microstructure and the spatial configuration of the matrixes of the snacks [36].

Taste could be improved by processes such as debittering, roasting, frying, fermentation and by adding flavourings, leghaemoglobin [33,53], and/or combining lupin with other foods such as shrimp [6].

The nutritional value can be improved by fortification with methionine [9], and by adding foods with sulphur-containing amino acids, i.e., eggs, fish, and/or cereals. The fat content could be reduced by using defatted lupin, by using hydrocolloids, or by replacing or combining frying with baking and/or vacuum frying [6].

In addition, sweet snacks should also be studied, because they might have enormous potential for being nutritionally improved [54,55,56]. Further, studies on the hypersensitivity of lupin snacks should also be conducted [57].

Finally, it is highly recommended that the amount of lupin cultivated worldwide be increased, as well as the production of lupin snacks.

## Data Availability

No new data were created or analyzed in this study. Data sharing is not applicable to this article.
